# Meta-Analysis of Structured Triglyceride versus Physical Mixture Medium- and Long-Chain Triglycerides for PN in Liver Resection Patients

**DOI:** 10.1155/2017/4920134

**Published:** 2017-08-28

**Authors:** Yajie Zhao, Chengfeng Wang

**Affiliations:** Department of Abdominal Surgical Oncology, National Cancer Center/Cancer Hospital, Chinese Academy of Medical Sciences and Peking Union Medical College, Beijing 100021, China

## Abstract

**Background:**

The use of total parenteral nutrition can affect liver function, causing a series of problems such as cholestasis. The aim of this meta-analysis was to compare structured triglyceride- (STG-) based lipid emulsions with physical medium-chain triglyceride (MCT)/long-chain triglyceride (LCT) mixtures in patients who had undergone liver surgery to identify any differences between these two types of parenteral nutrition.

**Methods:**

We searched the databases of PubMed, the Cochrane Library, Web of Science, EMBASE, and Chinese Biomedicine Database from January 2007 to March 2017 and included studies that compared STG-based lipid emulsions with physical MCT/LCT mixtures for surgical patients with liver disease.

**Conclusion:**

The STG was more beneficial than physical MCT/LCT on recovery of liver function and immune function. Therefore, STGs may represent a promising alternative to other types of lipid emulsions for hepatic surgery patients.

## 1. Introduction

Fat emulsion is an indispensable component of total parenteral nutrient (PN) preparations. Fat emulsions provide essential fatty acids and play an important role as an energy source. Furthermore, lipids are involved in the structure and function of cell membranes and receptors, gene expression modification, and inflammatory and immune response modification [1]. The liver is the main place where the metabolism of fat emulsions takes place. The use of total PN by patients undergoing long-term fasting can affect liver function, causing a series of problems such as cholestasis. However, the etiology of PN-associated hepatic injury remains unresolved. The use of fat emulsion for nutritional support among patients who have undergone liver surgery has caused controversy. However, in recent years, with the continuous development of fat emulsion technology and the deepening understanding of the mechanisms of hepatic metabolism, a new generation of fat emulsions has been created for patients who have undergone liver surgery. Structured triglyceride- (STG-) based lipid emulsions are composed of medium-chain fatty acids (MCFA) and long-chain fatty acids (LCFA) attached to the same glycerol skeleton. The production process involves heating the starting materials (medium-chain triglycerides [MCTs] and long-chain triglycerides [LCTs]) in the presence of enzyme catalysts to bring about restructuring. In this process, the triglyceride fatty acyl chains become randomized. The advantage of this process is the avoidance of the metabolism of physically mixed MCTs and LCTs, which interfere with each other. Many studies [2–4] have been performed to evaluate the effects of STG emulsions compared to physical MCT/LCT mixtures or LCT emulsions on hepatic integrity. In experimental settings, it has been repeatedly demonstrated that PN involving STG-based lipid emulsions is more beneficial than physical LCT/MCT mixtures. However, findings from studies involving surgical patients and those in intensive care units (ICUs) have yielded inconsistent outcomes. Chambrier and colleagues found no significant differences in hepatic function between patients who received STG emulsions or physical MCT/LCT mixtures, though only the differences in transaminases were assessed. To identify the optimal fat emulsion preparation, the effects on liver function impairment have to be clinically assessed, in addition to the ability of the fat emulsion to meet the patients' energy demands. The present meta-analysis was carried out to identify potential differences in nutritionally and clinically relevant endpoints among patients who have undergone gastrointestinal surgery and/or critically ill patients. Consequently, we systematically identified and reviewed the relevant evidence comparing STG-based lipid emulsions with physical MCT/LCT mixtures in patients with liver cancer who had undergone hepatectomy and we conducted a meta-analysis to identify potential differences between the two types of PN in terms of liver function, protein metabolism, and immune function.

## 2. Methods

**Figure 1:**
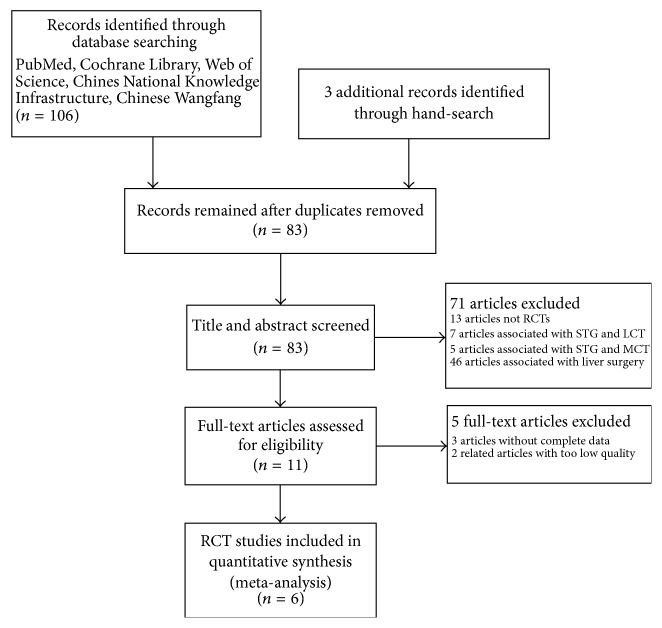


We searched both electronically and manually for journal articles published from January 2007 to March 2017. We searched PubMed, the Cochrane Library, Web of Science, EMBASE, and the Chinese Biomedicine Database using the following search terms: (structured triglyceride OR structured triacylglycerol OR structured lipid OR STG) AND (long-chain triglyceride OR long-chain triacylglycerol OR long-chain lipid OR medium-chain triglyceride OR medium-chain triacylglycerol OR medium-chain lipid OR MCT OR LCT OR MCT/LCT) AND (randomized controlled trial OR RCT). No language restriction was applied and the search was performed by two independent researchers (Figure 1).

### 2.1. Inclusion Criteria

The included studies met the following criteria: (1) the study compared STG emulsion with MCT/LCT mixture; (2) the study population consisted of patients with benign or malignant liver tumors who had undergone elective liver surgery; (3) the study was the latest publication (if the same data had been published multiple times); and (4) the study was a randomized controlled trial (RCT).

### 2.2. Exclusion Criteria

The following studies were excluded: (1) the PN type or the details of the surgical method were not reported; (2) the patients had not undergone liver surgery or had severe chronic liver disease (and were staying in an ICU); (3) there was no comparison of STG emulsion with MCT/LCT mixture; (4) the study outcomes did not include postoperative liver or immune function indicators; (5) the study was a report of data used in a later study; (6) low-quality studies: the quality of RCTs was evaluated based on the Jadad scale system: if the total score was <4, the RCT was deemed to be of low quality; (7) abstracts, case reports, letters, comments, and reviews without original data, and studies that presented insufficient data were not included; and (8) studies that were not RCTs were not included.

### 2.3. Literature Screening

All reports found during the literature search were screened by two independent investigators. When the two authors had a disagreement, they first tried to resolve it through discussion. If this failed, the final decision was made by a third author. EndNote reference management software was used to search for and remove any duplicate studies.

### 2.4. Data Extraction

The following data were independently extracted by the two investigators and checked by the other authors: title; authors; year of publication; country; study design; PN type; number of patients (by age and sex); postoperative liver function markers such as alanine aminotransferase (ALT), aspartate aminotransferase (AST), albumin, prealbumin, and total bilirubin; and postoperative immune function markers such as immunoglobulins A, M, and G (IgA, IgM, and IgG) and CD3+, CD4+, and CD8+ cell counts.

### 2.5. Statistical Analysis

RevMan (version 5.3.0) software provided by the Cochrane Collaboration was used to perform the meta-analysis in accordance with the Preferred Reporting Items for Systematic Reviews and Meta-Analyses (PRISMA) statement. Odds ratios (ORs) were used for the analyses of dichotomous variables and 95% confidence intervals (CIs) were reported. The Mantel-Haenszel, chi-square, and *I*^2^ tests were used to test the heterogeneity between the included studies. If *I*^2^ < 50%, this suggests that the heterogeneity is nonsignificant, and, consequently, a fixed-effects model should be used. If *I*^2^ > 50%, this suggests significant heterogeneity, and, consequently, a random-effects model should be applied. *P* < 0.05 was considered statistically significant. Funnel plots were used to assess any potential publication bias.

## 3. Results

### 3.1. Characteristics of the Included Studies, Quality Assessment, and Risk of Bias Assessment

**Figure 2:**
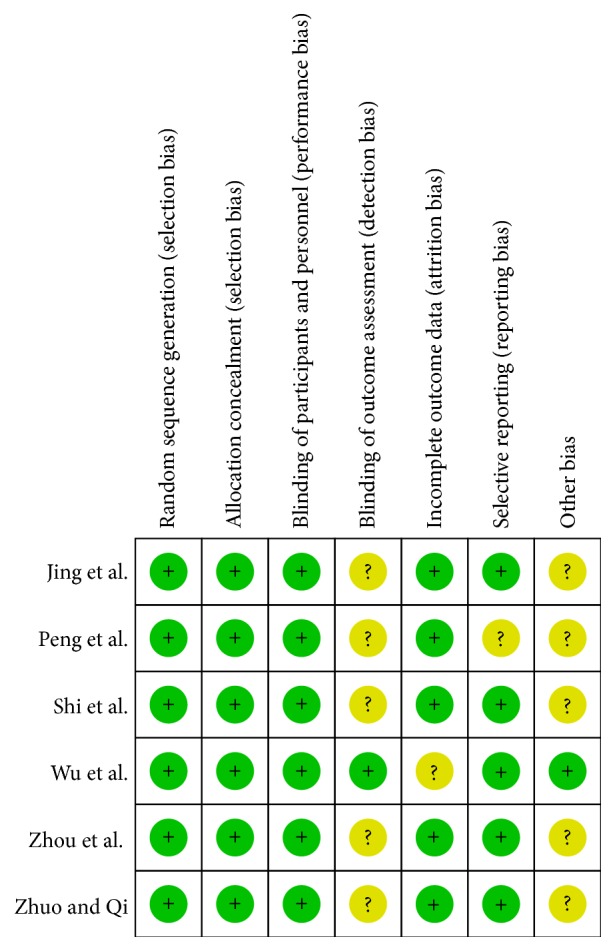


On the basis of the inclusion and exclusion criteria, six RCTs were included in the meta-analysis. The total number of patients was 516, of whom 259 were in the STG group and 257 were in the LCT/MCT group. The detailed characteristics of all the included studies are shown in Table 1. The quality of RCTs was evaluated based on the Jadad scale system, which was used to assess randomization, concealment of allocation, blinding, and withdrawals from the study. Each item was given a score of 0–2 and 7 score in total. If the total score was ≥4, the RCT was deemed to be of high quality; if the total score was <4, the RCT was deemed to be of low quality.  Figure 2 shows the results of the assessment of risk of bias. For the included RCTs, assessing the risk of bias involved six aspects (allocation concealment, incomplete outcome data, blinding, selective reporting bias, sequence generation, and other potential sources of bias) based on the quality checklist recommended in the Cochrane Handbook. In response to each item, “yes” indicates a low risk of bias; “unclear,” an uncertain risk of bias; and “no,” a high risk of bias. And all 6 studies included in the meta-analysis were reported from China, all were of relatively small, and that they all were of high quality.

### 3.2. Meta-Analysis Results

#### 3.2.1. Effect of STG Emulsions Compared to MCT/LCT Mixtures on Postoperative Liver Function

**Figure 3:**
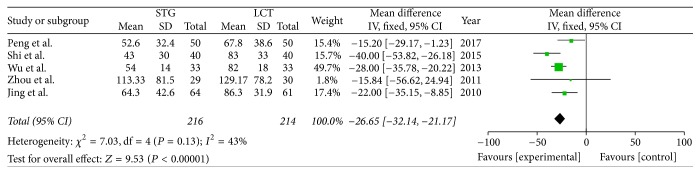
Meta-analysis of postoperative ALT.

*(1) Postoperative ALT*. Five of the included studies reported the ALT values on the fifth postoperative day, so we pooled the data from these studies to compare the STG and MCT/LCT groups. The results showed that there was a significant difference between the two groups (OR = −26.65; 95% CI, −32.14–−21.17; *P* < 0.00001; *I*^2^ = 43% for heterogeneity). Therefore, we used a fixed-effects model (Figure 3).

**Figure 4:**
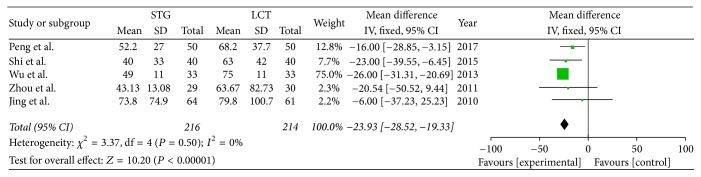
Meta-analysis of postoperative AST.

*(2) Postoperative AST. *Five of the included studies reported the AST values on the fifth postoperative day. The results of meta-analysis show that there is statistically difference between the two groups (OR = −23.93; 95% CI, −28.52–−19.33; *P* < 0.00001; *I*^2^ = 0% for heterogeneity). Therefore, we used a fixed-effects model (Figure 4).

**Figure 5:**
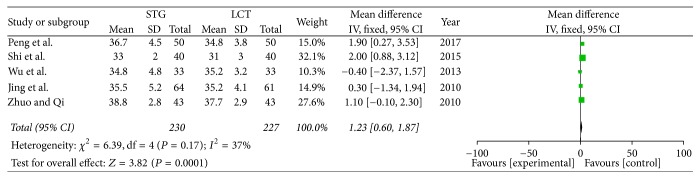
Meta-analysis of postoperative ALB.

*(3) Postoperative ALB. *Five of the included studies reported the ALB values on the fifth postoperative day. *I*^2^ (*I*^2^ = 37%) revealed no obvious heterogeneity among these studies. Therefore, we used a fixed-effects model. There was significant difference between two groups (OR = 1.23; 95% CI, 0.60–1.87; *P* = 0.001) (Figure 5).

**Figure 6:**
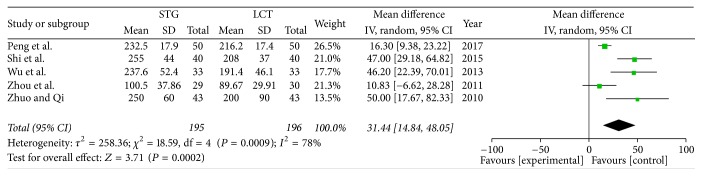
Meta-analysis of postoperative prealbumin.

*(4) Postoperative Prealbumin. *Five of the included studies reported the ALB values on the seventh postoperative day. The results of meta-analysis show that there is statistical difference between two groups (OR = 31.44; 95% CI, 14.84–48.05; *P* = 0.0002; *I*^2^ = 78% for heterogeneity). Therefore, we used a random-effects model (Figure 6).

**Figure 7:**
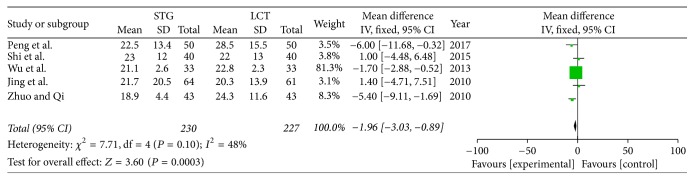
Meta-analysis of postoperative total bilirubin.

*(5) Postoperative Total Bilirubin. *Five of the included studies reported the total bilirubin values on the seventh postoperative day. *I*^2^ (*I*^2^ = 48%) revealed no obvious heterogeneity among these studies. Therefore, we used a fixed-effects model. There was significant difference between two groups (OR = −1.96; 95% CI, −3.03–−0.89; *P* = 0.0003) (Figure 7).

#### 3.2.2. Effect of STG Emulsions Compared to MCT/LCT Mixtures on Postoperative Immune Function

**Figure 8:**
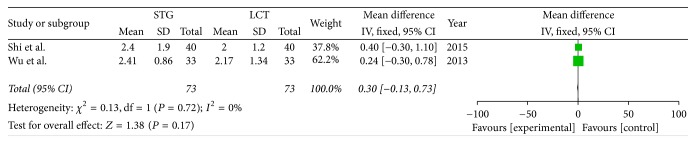
Meta-analysis of postoperative IgA.

**Figure 9:**
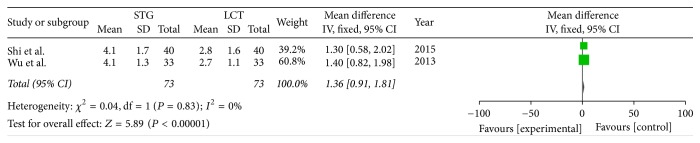
Meta-analysis of postoperative IgM.

**Figure 10:**
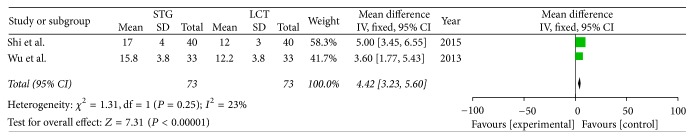
Meta-analysis of postoperative IgG.

**Figure 11:**
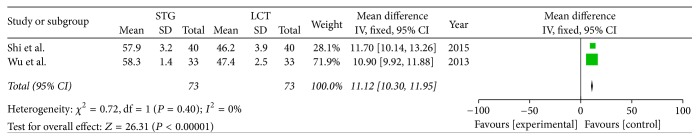
Meta-analysis of the postoperative CD3+ cell counts.

**Figure 12:**
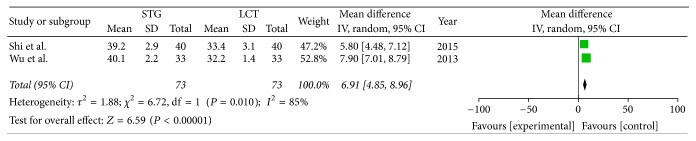
Meta-analysis of the postoperative CD4+ cell counts.

**Figure 13:**
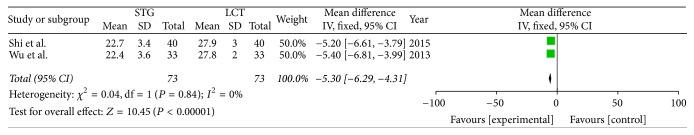
Meta-analysis of the postoperative CD8+ cell counts.

Two included studies reported the postoperative immune function. The results of meta-analysis show that there is a clear difference between the STG and MCT/LCT groups in terms of postoperative immunoglobulins M and G [IgM (OR = 1.36; 95% CI, 0.91–1.81; *P* < 0.00001; *I*^2^ = 0%) and IgG (OR = 4.42; 95% CI, 3.23–5.60; *P* < 0.00001; *I*^2^ = 23%)] and CD3+ (OR = 11.12; 95% CI, 10.30–11.95; *P* < 0.00001; *I*^2^ = 0%), CD4+ (OR = 6.91; 95% CI, 4.85–8.96; *P* < 0.00001; *I*^2^ = 85%), and CD8+ (OR = −5.30; 95% CI, −6.29–−4.31; *P* < 0.00001; *I*^2^ = 0%) cell counts but not in terms of immunoglobulin A (IgA) (OR = 0.30; 95% CI, −0.13–0.73; *P* = 0.17; *I*^2^ = 0%) (Figures 8–13).

### 3.3. Publication Bias

**Figure 14:**
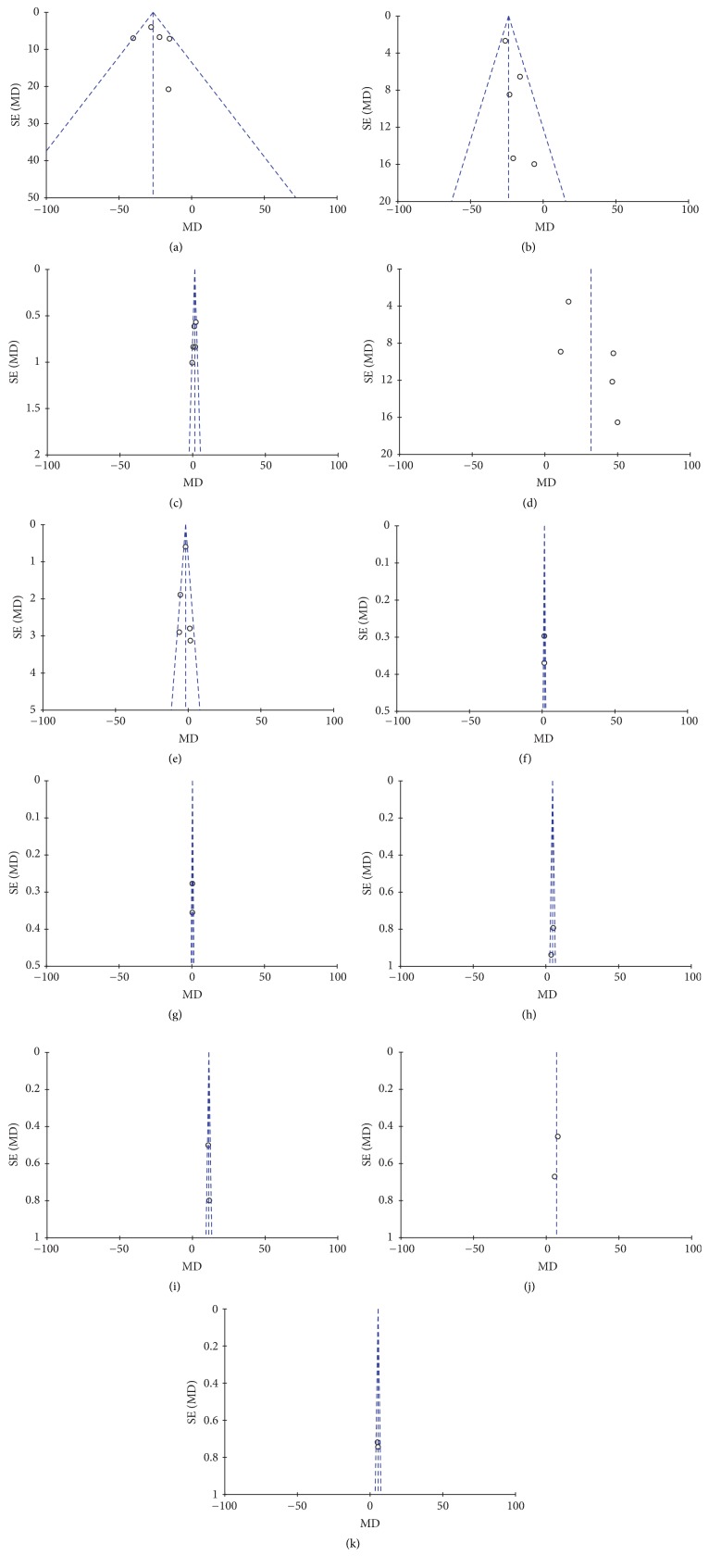
Funnel plots: (a) ALT; (b) AST; (c) albumin; (d) prealbumin; (e) total bilirubin; (f) IgA; (g) IgM; (h) IgG; (i) CD3+; (j) CD4+; (k) CD8+.

Funnel plots were constructed to assess the publication bias in this meta-analysis. As shown in Figure 14, there was no evident asymmetry in the funnel plots, indicating a low probability of publication bias.

## 4. Discussion

Dietary lipids are important factors that affect xenobiotic metabolism. Hepatic microsomal cytochrome P450 plays a key role in the metabolism of various endogenous and exogenous compounds. Consequently, total PN (which includes lipids) may be associated with liver disease, which frequently occurs in neonates and in patients with liver disease.

STG emulsions are an alternative to physical MCT/LCT mixtures. STG emulsions can be manufactured by mixing MCT and LCT oils and heating the mixture in the presence of a catalyst. A number of pathologic changes leading to the development of hepatic dysfunction have been observed in PN patients [11]. The mechanism behind PN-related liver dysfunction remains largely unknown, but it is likely to be multifactorial.

The main results of the present study showed that STG emulsions have no effect on hepatocellular integrity, whereas both physical MCT/LCT mixtures cause subclinical hepatic injury in postoperative patients [12]. The meta-analysis results indicated that there were significant differences between the STG and MCT/LCT groups in terms of postoperative liver function markers such as AIL, AST, albumin, prealbumin, and total bilirubin. The results showed that the levels of postoperative albumin and prealbumin in the STG group were higher than in the MCT/LCT group. In contrast, the levels of ALT, AST, and total bilirubin on the seventh postoperative day were lower in the STG compared to the MCT/LCT group.

SGT emulsions did not increase the burden on the liver and they were beneficial in terms of inhibiting protein decomposition, reducing tissue consumption, and improving the metabolism of human body protein. This may be related to the relatively stable nature of STGs. STG emulsions did not increase the rate of cholestasis and they led to benefits in terms of decreasing the levels of postoperative total bilirubin. Total bilirubin can reflect postoperative liver function and biliary obstruction. Sandstrom and colleagues [13] verified that STGs are oxidized more rapidly than LCTs, probably secondary to their rapid clearance from the plasma compartment and better availability for oxidative processes. This mechanism may be important in nutrition strategies such as PN. Kruimel and colleagues [14] demonstrated that there was a faster clearance of STGs compared with a physical MCT/LCT mixture, verified by an improvement in the nitrogen balance.

The meta-analysis results also showed that there was a clear difference between the STG and MCT/LCT groups in terms of postoperative immune function. IgG, IgA, and IgM levels are common indicators that can reflect humoral immune function. The level of CD3+ T cells reflects the overall level of cellular immunity. CD4+ T cells can promote B cell differentiation (to induce the production of antibodies), activate other cells so that they secrete lymphatic factor, and play a role in inflammatory reactions and a mediating role. CD8+ T cells are a kind of immune suppression cell, as they inhibit antibody secretion and T-cell proliferation.

The results indicated that STG emulsions can improve cellular and humoral immune function. Several clinical and experimental studies [15] have suggested that while fat emulsion provides essential fatty acids and energy, it can impair immune function and increase the risk of infection, and the mechanism by which it does this is mainly related to fatty acids. Fatty acids are an important component of cell membranes, and the carbon chain length and saturation of the fatty acids in cell membranes play important roles in immune cell interactions. Fatty acids affect immune responses mainly via structural change (i.e., changes in the fatty acid composition of the phospholipids in cell membranes) and chemical mediation. Chemical mediation can affect the synthesis and release of factors such as interleukin 1 (IL-1) and tumor necrosis factor (TNF). IL-1 and TNF are important immune regulators that are necessary for the production of B cells, T cells, and nonspecific immune T cells.

The immune function inhibition caused by some fat emulsions is mainly related to LCTs, as the fatty acid carbon chains of MCTs do not contain unsaturated double bonds, so MCTs are chemically stable and easily undergo peroxidation. MCTs can be cleared directly and quickly (they are not readily deposited in the liver, lungs, and reticuloendothelial system), and esterification does not readily occur, which can reduce the system load.

During the metabolism of STG emulsions, the MCTs and LCTs are released in a 1 : 1 ratio, so using an STG emulsion does not lead to the accumulation of LCTs resulting from the MCT that is easily metabolized. MCT metabolism does not produce arachidonic acid, which has an inhibitory effect on the immune system. In addition, ketone bodies, which are produced during MCT metabolism, can promote the proliferation of immune and intestinal mucosal epithelial cells [16].

Several RCTs showed that, among patients receiving MCT/LCT mixtures, serum IgG, IgM, CD3+, and CD4+ levels dropped significantly by the fifth postoperative day, with no significant recovery by the seventh day. In contrast, in patients receiving an STG emulsion, the change in serum IgG, IgM, CD3+, and CD4+ levels was not very obvious by the fifth postoperative day, and these values recovered (to preoperative levels) by the seventh day [17]. This indicates that STG emulsions do not impair immune function and that they may be effective for the recovery of postoperative immune function in patients with liver cancer. The effect may also be related to the improvement of the patients' nutritional status.

A major limitation of the study was that it only included a small number of high-quality RCTs, which are all coming from the same country, China. Another potential limitation is that some of the RCTs used epidural analgesia so we were unable to exclude its influence on hepatic blood flow (which can affect liver function). However, the type of analgesia did not differ between study groups in any of the RCTs, so any influence on our results is less likely. However, the different types of liver-protecting therapy used in the RCTs may have affected their outcomes and increased the heterogeneity between the included studies.

## 5. Conclusions

Compared to physical MCT/LCT mixtures, STG emulsions can safely improve nutritional status while relieving the burden associated with liver metabolism and inflammatory reactions in patients with liver carcinoma after hepatectomy. The meta-analysis showed that STG emulsions were more beneficial than MCT/LCT mixtures in terms of recovery of liver function and immune function. Therefore, STG emulsions represent a promising alternative to other types of lipid emulsion for liver surgery patients. However, large multicenter RCTs are needed to better evaluate the most preferable PN strategies. In addition, further studies are needed to investigate the clinical relevance of the effects of PN strategies, particularly in patients requiring long-term PN, those with preexisting liver injuries, and those undergoing surgery that affects liver function, for example, hemihepatectomy. In summary, PN involving physical MCT/LCT mixtures is likely to cause subclinical hepatic injury, whereas STG emulsions have no detectable effect on hepatocellular integrity.

## Figures and Tables

**Table 1:** The characteristics of all the included studies.

Author	Year	Country	Study type	Group	Patients number	Male/female	Age, y	Study quality RCT (Jadad system) Retro (NOS system)
Peng et al. [5]	2017	China	RCT	STG	50	33/17	52.1 ± 8.5	7
				MCT/LCT	50	30/20	51.3 ± 3.2	
Shi et al. [6]	2015	China	RCT	STG	40	25/15	56 ± 10	5
				MCT/LCT	40	26/14	57 ± 11	
Wu et al. [7]	2013	China	RCT	STG	33	25/8	49.1 ± 1.8	7
				MCT/LCT	33	25/8	51.3 ± 3.2	
Zhou et al. [8]	2011	China	RCT	STG	29	24/5	53.83 ± 10.29	7
				MCT/LCT	30	23/7	52.37 ± 12.27	
Jing et al. [9]	2010	China	RCT	STG	64	47/17	49.9 ± 8.6	5
				MCT/LCT	61	45/16	48.7 ± 10.5	
Zhuo and Qi [10]	2010	China	RCT	STG	43	25/18	43.0 ± 5.0	7
				MCT/LCT	43	27/16	45.0 ± 8.0	

RCT = randomized controlled trial; STG: structured triglycerides; MCT/LCT: medium- and long-chain triglycerides.

## References

[1] Elia M., Austin P., Stratton RJ., Sobotka L. (2011). Indications for nutritional support. *Basics in Clinical Nutrition*.

[2] Calder P. C., Jensen G. L., Koletzko B. V., Singer P., Wanten G. J. A. (2010). Lipid emulsions in parenteral nutrition of intensive care patients: current thinking and future directions. *Intensive Care Medicine*.

[3] Ott J., Hiesgen C., Mayer K. (2011). Lipids in critical care medicine. *Prostaglandins Leukotrienes and Essential Fatty Acids*.

[4] Wanten G. J., Naber A. H. (2007). Immune modulation by parenteral lipid emulsions. *The American Journal of Clinical Nutrition*.

[5] Peng N., Lyu F., Guo Q. (2017). Efficacy comparison of structured fat emulsion and medium/long chain fat emulsion for nutritional support in liver carcinoma patients after hepatectomy. *China Pharmacy*.

[6] Shi X. L., Wang S., Wu Y. F. (2015). Effects of structured triglyceride on postoperative recovery of patients with primary liver cancer after hepatectomy: a prospective study. *Chinese Journal of Digestive Diseases*.

[7] Wu Z. S., Zhang F., Wu X. F. (2013). Effects of structured lipid emulsion on protein metabolism and immunologic function in patients with liver cancer. *Jiangsu Medical Journal*.

[8] Zhou J., Ying W. D., Xu G. L. (2011). The effect of different triacylglycerols on postoperative PN for patients with primary liver cancer after hepatectomy. *Chinese Journal of General Surgery*.

[9] Jing K., Zhang Z. W., Huang Z. Y. (2010). The effect of different triacylglycerols in liver and renal functions of hepatotectomy patient postoperatively. *Chinese Journal of Experimental Surgery*.

[10] Zhuo D. Q., Qi P. (2010). Influence of structured triglyceride on the surgical patients with liver disease. *Chinese Journal of Experimental Surgery*.

[11] Chambrier C., Lauverjat M., Bouletreau P. (2006). Structured trig: lyceride emulsions in parenteral nutrition. *Nutrition in Clinical Practice*.

[12] Larbi A., Grenier A., Frisch F. (2005). Acute in vivo elevation of intravascular triacylglyeerol lipolysis impairs peripheral T cell activation in humans. *The American Journal of Clinical Nutrition*.

[13] Sandstrom R., Hyltander A., Korner U., Lundholm K. (1995). Structured triglycerides were well tolerated and induced increased whole body fat oxidation compared with long-chain triglycerides in postoperative patients. *Journal of Parenteral and Enteral Nutrition*.

[14] Kruimel J. W., Naber T. H., Van Der Vliet J. A., Carneheim C., Katan M. B., Jansen J. B. (2001). Parenteral structured triglyceride emulsion improves nitrogen balance and is cleared faster from the blood in moderately catabolic patients. *Journal of Parenteral and Enteral Nutrition*.

[15] Lin M.-T., Yeh S.-L., Tsou S.-S., Wang M.-Y., Chen W.-J. (2009). Effects of parenteral structured lipid emulsion on modulating the inflammatory response in rats undergoing a total gastrectomy. *Nutrition*.

[16] Chambrier C., Lauverjat M., Bouletreau P. (2006). Structured Triglyceride Emulsions in Parenteral Nutrition. *Nutrition in Clinical Practice*.

[17] Dybul M., Nies-Kraske E., Daucher M. (2003). Long-cycle structured intermittent versus continuous highly active antiretroviral therapy for the treatment of chronic infection with human immunodeficiency virus: effects on drug toxicity and on immunologic and virologic parameters. *Journal of Infectious Diseases*.

